# Deferasirox associated with liver failure and death in a sickle cell anemia patient homozygous for the −1774delG polymorphism in the *Abcc2* gene

**DOI:** 10.1002/ccr3.1040

**Published:** 2017-06-15

**Authors:** Caroline C. B. Braga, Bruno Deltreggia Benites, Dulcineia M. de Albuquerque, Marisa C. Alvarez, Tiago Seva‐Pereira, Bruno K. L. Duarte, Fernando F. Costa, Simone C. O. Gilli, Sara T. O. Saad

**Affiliations:** ^1^ Hematology and Transfusion Medicine Center University of Campinas Campinas Brazil; ^2^ Unit of Liver Transplantation University of Campinas Campinas Brazil

**Keywords:** Abcc2, Deferasirox, liver failure, polymorphisms, sickle cell disease

## Abstract

This manuscript describes the case of a patient with sickle cell anemia who died of fulminant hepatitis after therapy with the iron chelator Deferasirox. The patient was homozygous for the −1774delG polymorphism in the *Abcc2* gene, which raises the concern about the use of hepatotoxic drugs in this specific context.

## Introduction

The increase in life expectancy of patients with sickle cell disease (SCD) has led to the need for constant revisions of their clinical approach. Blood transfusions are part of the treatment of these patients and provide many benefits such as preventing and treating acute and chronic events; however, iron overload is a major complication 1, 2, 3. The use of iron chelators especially in this group of patients may present some obstacles with quite deleterious effects 4, 5, 6. The use of biological markers that could define groups at greater risk for these complications would help to define the best chelator for a particular patient 7.

Multidrug resistance protein 2 (MRP2) is a member of the ATP‐binding cassette (ABC) transporter family of membrane proteins encoded by the *Abcc2* gene and is expressed in the canalicular part of the hepatocyte. MRP2 acts upon biliary transport of several endogenous compounds and many drugs, including the iron chelator Deferasirox 8. Thus, genetic polymorphisms related to the *Abcc2* gene, which encodes the MRP2 protein, may influence individual susceptibility to hepatotoxicity related to Deferasirox. Patients presenting with −24C>T and/or −1774delG *Abcc2* gene polymorphisms have been previously shown to be at a higher risk of hepatotoxicity than those with the wild‐type allele 9.

We conducted a genetic sequencing study of these polymorphisms in 15 patients with sickle cell disease and hemochromatosis receiving Deferasirox (DFX), and observed that four patients (26.6%) developed hepatotoxicity: One (25%) had the rs717620 (−24C>T) polymorphism in heterozygosity; three (75%), however, had no polymorphism in any of their alleles and had hepatotoxicity (OR = 3.02, 95% CI = 0.03–286.42, *P* = 0.47). Moreover, considering the four patients who presented hepatotoxicity, only one (25%) had the homozygous −17774delG polymorphism and three (75%) of these patients despite having no polymorphism still experienced hepatotoxicity (OR = ∞, 95% CI = 0.07 to ∞, *P* = 0.26). Interestingly, this polymorphism was present in homozygosis in one patient with sickle cell anemia who suffered fulminant hepatitis leading to death during drug therapy.

## Case History

We present the case of a 43‐year‐old female with sickle cell anemia undergoing chronic blood transfusions due to severe anemia, refractory to hydroxyurea, and erythropoietin, who started 20 mg/kg/day oral DFX therapy in January 2014. Despite having received Deferoxamine for several years, chelation was not effective due to low compliance. She had no cardiac iron deposits (cardiac T2* measured by MRI was 30.8 msec; reference value >20 msec); however, the liver iron concentration, assessed by MRI, was high, with an LIC of 4.1 mg/g. Before initiating DFX therapy, hemoglobin was 8.9 g/dL (11.8–14.8 g/dL); white blood cell count, 10.6 × 103/*μ*L (3.9–11.1 × 103/*μ*L), Hemoglobin S was 17%, alkaline phosphatase (ALP), 60 U/L (35–104 U/L); *γ*‐glutamyltransferase (GGT), 181 U/L (<40 U/L); ALT, 26 U/L (<33 U/L); AST, 37 U/L (<32 U/L); LDH, 592 U/L (<480 U/L); ferritin, 3208 ng/mL (13–150 U/L) and transferrin saturation index was 79%. Hepatitis B serology showed positive anti‐HBs and anti‐HBc with negative HBsAg, HBeAg anti‐HBe. Hepatitis C antibody (anti‐HCV) was positive; however, HCV‐RNA was not detected by real‐time PCR assay, and thus, the patient was not considered to suffer from chronic hepatitis C. Abdominal ultrasound assessment showed no signs of chronic liver disease. Autoantibodies for autoimmune hepatitis (antinuclear antibody – ANA, liver and kidney microsome type 1 antibody – anti‐LKM1, and smooth muscle antibody – SMA) were negative.

**Figure 1:**
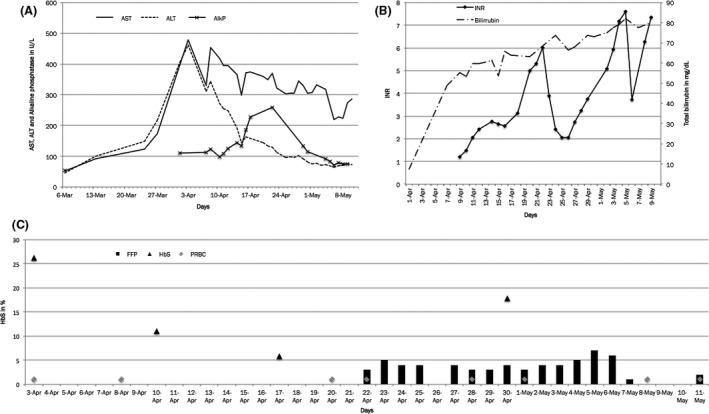
Laboratory parameters and their evolution over the period of patient evaluation. (A) AST, ALT, Alkaline Phosphatase; (B) INR; (C) HbS in %.

The patient had no history of liver or pulmonary sequestration, no painful crises, nor did she smoke, drink, or use illicit drugs. Her usual medications were folate, enalapril, alendronate, and CaCO3 + Vit. D. At physical examination, liver was palpable 2 cm below the right costal margin. During DFX therapy, serum urea and creatinine levels and liver function were monitored weekly. She initially showed a good tolerance to the medication, with no report of adverse effects, maintaining normal renal function as well as parameters of liver function. However, 56 days after the beginning of DFX therapy, transaminases levels increased 4X the ULN and the drug was immediately discontinued (Figure 1). After DFX discontinuation, AST and ALT levels presented progressive elevation, and bilirubin levels were also markedly increased. A presumable diagnosis of hepatocellular drug‐induced liver injury (DILI) was performed, and the injury was initially classified as moderate (score 2) according to the Drug‐Induced Liver Injury Network criteria. The patient was then admitted to our inpatient unit for further investigation and monitoring.

The patient underwent an abdominal ultrasound assessment that showed no abnormalities, and a Doppler ultrasound which showed no signs of thrombosis. All her medications were withdrawn, and an intensification of transfusion therapy was adopted to maintain HbS below 20%. Despite these measures, the patient kept increasing transaminases and bilirubin levels. Alkaline phosphatase and gama‐glutamyltransferase levels also increased; however, their elevation was disproportionally lower than the bilirubin levels (Fig. 1). Thirteen days after patient admission, her liver function deteriorated as demonstrated by a prolonged prothrombin time (PT), with minor bleeding, prompting the need of fresh‐frozen plasma transfusions (FFP) (Fig. 1). On the next day, she spiked a fever, which was attributed to an infected perimalleolar ulcer, and antibiotics were administered (Clindamycin and Ceftriaxone).

The patient continued to show bleeding, progressive in intensity, requiring larger volumes of FFP, due to unresponsive prolonged PTs. On the 26th day at the inpatient clinic, she presented another episode of fever, with no clear signs or symptoms of infection, which led to a change in her antibiotics to piperacillin–tazobactam. Five days after that, she presented with grade III/IV encephalopathy, and a rapid clinical deterioration characterized by a refractory distributive shock and acute renal failure requiring dialysis, which evolved to multi‐organ failure, in <24 h, followed by cardiac arrest and death. These events are summarized in Figure 1.

## Discussion

We have described what is, to the best of our knowledge, a case of probable liver failure‐related death associated with Deferasirox. Liver injury occurred in close temporal proximity to drug use, and other causes of liver damage were excluded. The drug is not referenced in the FDA Liver Toxicity Knowledge Base; however, according to the Council for International Organizations of Medical Sciences/Roussel Uclaf Causality Assessment Method (CIOMS/ RUCAM) scale 10, the case herein reported scored six points (time from drug intake until reaction onset: +2, exclusion of non‐drug‐related causes: +2, previous information on hepatotoxicity: +2), indicating a probable Deferasirox‐induced hepatotoxicity. This scale is widely used in cases of liver injury and was designed for idiosyncratic drug‐induced liver injury (DILI) causality assessment through several domains, including chronological relationship between drug exposure and hepatic injury. Nevertheless, using the scale of Naranjo et al., a score of five was calculated for the case herein described (existence of previous reports on the reaction: +1, adverse event appeared after the drug was administered: +2, and no other causes for the reaction: +2), indicating a probable adverse drug reaction 11. Lowering the patient's HbS levels through exchange transfusion did not prevent a further decline of liver function, reinforcing the idea that the presence of −17774delG polymorphism in homozygosis may have played a role in this outcome rather than SCD itself.

Our study highlights the concern regarding the use of hepatotoxic drugs in patients with sickle cell anemia and the need for markers of possible hepatotoxicity that could indicate, which patients are eligible for the safe use of these drugs.

## Authorship

SCOG and BDB: designed the research study. CCBB, DMA, and MCA: performed the research. BDB, SCOG, and CCBB: wrote the article. TSP, BKLD, and FFC: cooperated in the analysis of the results. STOS: coordinated the research.

## Conflict of Interest

The authors declare no conflict of interests.
